# Genomic surveillance of TB: cluster identification and transmission insights

**DOI:** 10.5588/ijtldopen.26.0115

**Published:** 2026-11-13

**Authors:** E. Commiesie, R. Anthony, F. Gopie, M. Kamst, T. Koendjbiharie, S. Sewdien, D. Stijnberg, S. Vreden, R. de Zwaan, G. de Vries

**Affiliations:** 1 National TB Program, Paramaribo, Suriname;; 2 Anton de Kom University of Suriname, Paramaribo, Suriname;; 3 National Institute for Public Health and the Environment, Bilthoven, the Netherlands;; 4 Academic Hospital Paramaribo, Paramaribo, Suriname;; 5 Bureau of Public Health Suriname, Paramaribo, Suriname.

**Keywords:** tuberculosis, Suriname, epidemiology, whole-genome sequencing, transmission, TB cluster

## Abstract

**INTRODUCTION:**

TB incidence in Suriname has remained stable over the last decade. Understanding transmission patterns is essential for developing effective TB control strategies to interrupt ongoing transmission.

**METHODS:**

We conducted a descriptive analysis of routinely collected demographic and clinical data combined with whole-genome sequencing (WGS) data. Patients were classified into large (>6 patients) clusters, small (2–6 patients) clusters, or as non-clustered cases (unique). Bivariate and multivariable analyses compared patients in large clusters with those in small clusters and non-clustered patients. Epidemiological links obtained through patient interviews were compared with WGS findings.

**RESULTS:**

Of 698 TB patients notified during 2019–2024, WGS data were available for 368 (53%); 85% belonged to a genomic cluster. Indigenous ethnicity was inversely associated with membership in a large versus a small cluster (adjusted odds ratio [aOR] = 0.3; 95% confidence interval [CI] = 0.1–0.6). Compared with non-clustered cases, patients in large clusters were more likely to report illicit drug use (aOR = 3.0; 95% CI = 1.1–8.9).

**CONCLUSION:**

WGS provided detailed insights into *Mycobacterium tuberculosis* transmission in Suriname. Illicit drug use was associated with membership in large transmission clusters, highlighting the need for early case finding and strengthened collaboration between TB and substance-use services.

TB remains a major public health threat, affecting an estimated 10.7 million people in 2024.^1^ The WHO End TB Strategy aims to reduce TB incidence in 2035 by 90% compared to 2015.^2^ The global incidence however had dropped only by 12% in 2024, far below the 2025 target of 50%.^1^ This slow progress underscores the need to better understand the drivers and challenges of TB transmission in order to effectively interrupt ongoing spread.

Suriname is located on the northeastern coast of South America with an estimated population of 626,028 people in 2023. Administratively, the country is divided into 10 districts, with the urban districts (Paramaribo and Wanica) accounting for approximately 70% of the population. Suriname has a multi-ethnic population comprising 27% Hindustani, 22% Maroon, 16% Creole, 14% Javanese, 4% Indigenous population, and 17% others.^3^ The TB incidence in Suriname was 34 per 100,000 population in 2024 and has remained nearly unchanged compared to 2015 (35 per 100,000).^1^ The HIV prevalence among individuals aged 15–49 years was 1.6% in 2023.^4^

Whole-genome sequencing (WGS) is a molecular technique that determines the DNA sequence of *Mycobacterium tuberculosis*, enabling the identification of lineages, resistance patterns, and genetic relatedness between strains.^5,6^ WGS can therefore detect molecular clusters and potential outbreaks.^6,7^ Large clusters are of particular public health concern because they indicate ongoing transmission and can guide targeted control measures.^8^ Several conditions need to be met to use WGS for epidemiological surveillance, including sufficiently high percentage of cases sequenced, sustained implementation over multiple years, and integration with conventional epidemiological investigations.^9–11^ In Suriname, WGS was first implemented in 2018 for rifampicin-resistant TB.^12^ Since 2019, all *M. tuberculosis* isolates have been sent to the National Institute for Public Health and the Environment (RIVM) in the Netherlands for WGS-based typing.

The aim of this study is to analyse the WGS-based TB clusters in Suriname over a 6-year period, to better understand the cluster structure and risk factors for transmission in the country, and identify opportunities to improve TB control.

## METHODS

TB cases are routinely notified to the National Tuberculosis Programme (NTP). As part of routine surveillance, NTP nurses collect demographic, social, and clinical information for all patients and record these data in the National TB Register. All notified TB cases from 2019 to 2024 were included in this study; recurrent episodes occurring within this period were excluded. The following variables were extracted from the National TB Register: year of notification, sex, age, nationality, ethnicity, urban residence, case type, disease type, Xpert MTB/RIF results, culture and drug-susceptibility test results, HIV status, use of illicit drugs (e.g., marihuana or cocaine), and presence of diabetes mellitus. WGS data generated by the RIVM are routinely added to the National TB Register and were included in the analysis.

### WGS and clusters

At the Central Laboratory of Suriname, Löwenstein–Jensen culture was used for the culturing of *M. tuberculosis* from diagnostic samples from presumptive TB patients. Culture-positive samples were heat-killed (20 min at 100°C) and sent in quarterly batches to the RIVM for WGS. At RIVM, DNA was extracted from the lysates by mechanical lysis and column purification,^13^ sequenced using Illumina technology and SNP variability detected after mapping to a reference genome using the RIVM in-house pipeline.^14^ Sequencing results were reported back to both the Central Laboratory and the NTP. The average turnaround time, comprising shipment of lysates to RIVM (approximately 7 days), DNA extraction and sequencing (7–10 days), and reporting results, was approximately 14–17 days. Phylogenetic diversity among isolates was assessed using a validated 62-single-nucleotide polymorphisms (SNPs) panel described by Coll et al., which enables classification of *M. tuberculosis* complex isolates into 7 major lineages and 55 sub-lineages.^15^ An isolate was considered to belong to the same cluster if it differed ≤12 SNPs from an isolate from another individual.^16,17^ Isolates with more SNPs difference were classified as unique. Clusters were further categorised by size, as small clusters (2–6 patients) and large clusters (>6 patients), based on empirical distribution of cluster size. Drug resistance was predicted from the WGS data using the WHO Catalogue of mutations in *M. tuberculosis* complex.^18^

### Epidemiological links

TB nurses routinely ask newly diagnosed TB patients the question, ‘Do you know anyone with TB?’ to identify a presumed index patient. This information is recorded in paper-based patient files but is not routinely entered into the National TB Register. We reviewed all available patient files, extracted information on presumed index patients, and added these data to the study database. We verified if the presumed index patients were recorded in the electronic National TB Register, which has been operational since 2010. For patients with a confirmed match, we calculated the median time interval between treatment start dates of patients and their presumed index cases. Epidemiological links identified through patient interviews were compared with genetic links identified by WGS. For genetically confirmed pairs, the median time interval between treatment start dates was calculated as described above. To assess the proportion of clustered cases that were epidemiologically linked to another case within the same cluster, we applied the *n*–1 method, that is, excluding the first case in each cluster.^12^ This analysis was stratified by cluster size (small vs. large clusters).

### Statistical analysis

All variables were categorical and are presented as absolute numbers and relative frequencies. A χ² test was used to compare the distribution of factors between patients in large clusters versus small clusters, and patients in large clusters versus patients with unique WGS profiles. Bivariate analyses were performed to estimate the odds ratios (ORs) and 95% confidence intervals (CIs) for both comparisons. Variables with a *P* value < 0.25 in bivariate analysis were included in the multivariable logistic regression models. Statistical significance was defined as a *P* < 0.05.

### Ethical statement

Ethical approval for this study was received from the Human Scientific Research Ethic Committee of the Ministry of Health of Suriname.

## RESULTS

Between 2019 and 2024, the National Tuberculosis Programme notified 730 TB episodes, of which 72 (10%) occurred in patients previously treated for TB. Twenty-eight persons were notified twice (two TB episodes) during the study period, and two persons three times (three episodes). These second and third episodes were excluded from the analysis, resulting in a final study population of 698 unique individuals (Figure).

**Figure.**
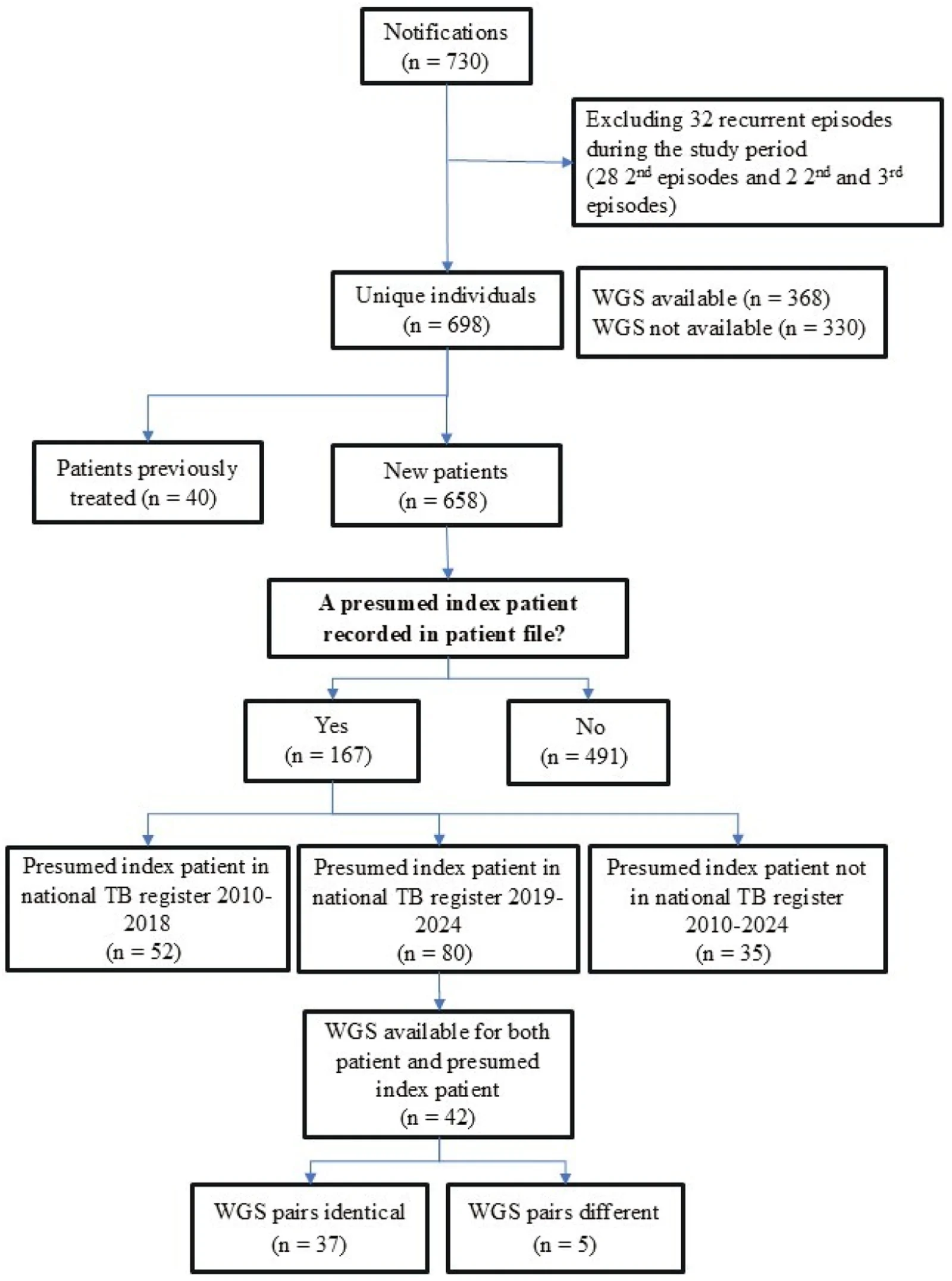
Flow chart of TB patients in Suriname, 2019–2024, included in the study, with whole-genome sequencing (WGS) results and information on presumed epidemiological link.

Ninety-four percent of TB patients had the Surinamese nationality (Table 1). The largest ethnic group among patients was Creole (34%), followed by Maroon (17%). The Indigenous population contributed 11% to the number of TB patients. The majority of patients (77%) resided in the two urban districts of Suriname. HIV, illicit drug use, and diabetes mellitus were reported in 17%, 21%, and 16% of TB patients, respectively. WGS results were available for 368 of the patients, which was 96% of the culture positive (and 53% of all the patients).

**Table 1.** Demographic and disease characteristics of TB patients with and without whole-genome sequencing (WGS) results in Suriname, 2019–2024.

Characteristics	Value	Patients with WGS		Patients without WGS		Total patients
		(N = 368)		(N = 330)		(N = 698)
		n	%	n	%	n
Year	2019	69	50%	70	50%	139
	2020	52	51%	50	49%	102
	2021	30	35%	56	65%	86
	2022	80	63%	46	37%	126
	2023	75	63%	45	38%	120
	2024	62	50%	63	50%	125
Sex	Female	96	49%	100	51%	196
	Male	272	54%	230	46%	502
Age (years)	<25	44	39%	68	61%	112
	25–44	145	59%	102	41%	247
	45–64	152	54%	128	46%	280
	≥65	27	46%	32	54%	59
Nationality	Surinamese	347	53%	312	47%	659
	Non-Surinamese	21	54%	18	46%	39
Ethnicity	Creole	120	51%	116	49%	236
	Maroon	51	44%	66	56%	117
	Hindustani	55	56%	44	44%	99
	Indigenous	50	64%	28	36%	78
	Javanese	43	67%	21	33%	64
	Other	49	47%	55	53%	104
Residency	Urban	275	51%	261	49%	536
	Other	93	57%	69	43%	162
HIV test result	Negative	313	57%	232	43%	545
	Positive	51	42%	71	58%	122
	Unknown	4	13%	27	87%	31
Illicit drug use	No	266	52%	243	48%	509
	Yes	93	63%	55	37%	148
	Unknown	9	22%	32	78%	41
Diabetes mellitus	No	294	53%	266	48%	560
	Yes	67	59%	46	41%	113
	Unknown	7	28%	18	72%	25
Case type	New	348	53%	310	47%	658
	Previously treated	20	50%	20	50%	40
Disease type	Pulmonary	360	60%	242	40%	602
	Extra-pulmonary	8	8%	88	92%	96
Bacteriology	Xpert MTB/RIF positive	349	67%	173	33%	522
	Culture positive	368	96%	14	4%	382
	RIF resistance	49	78%	14	22%	63

The total number of notifications in 2019–2024 was 730. Patients with a second and third disease episode during the study period (n = 32) were excluded.

Using the Coll lineage classification method, 26% of the 368 patients with a WGS result were attributed to lineage 1 strains, 10% to lineage 2, 1% to lineage 3, and 62% to lineage 4 (see Supplementary Tables 1 and 2). The Coll method further classified the isolates into 22 sub-lineages, while WGS identified 83 distinct genomic profiles. Overall, 66% of patients (n = 244) belonged to six large WGS clusters (Table 2), consisting of 98 (26%), 51 (14%), 43 (12%), 22 (6%), 19 (5%), and 11 (3%) cases, respectively. Each large cluster had a different sub-lineage, except for two clusters assigned to lineage 4.8; however, the pairwise SNP distance between these two clusters exceeded 200 SNPs. Seventy patients (19%) belonged to small clusters: one cluster of six cases, one of five cases, six clusters of four cases, five clusters of three cases, and ten clusters of two cases. The remaining 54 patients (15%) had unique WGS profiles which did not cluster with any other isolate.

**Table 2.** Demographic and disease characteristics of TB patients with whole-genome sequencing (WGS) results according to cluster size in Suriname, 2019–2024.

Characteristics	Value	Cases in large WGS clusters		Cases in small WGS clusters		Cases with unique WGS		Total patients with WGS
		(N = 244)		(N = 70)		(N = 54)		(N = 368)
		n	%	n	%	n	%	n
Sex	Female	58	60%	18	19%	20	21%	96
	Male	186	68%	52	19%	34	13%	272
Age (years)	<25	28	64%	13	30%	3	7%	44
	25–44	98	68%	31	21%	16	11%	145
	45–64	104	68%	21	14%	27	18%	152
	≥65	14	52%	5	19%	8	30%	27
Nationality	Surinamese	236	68%	66	19%	45	13%	347
	Non-Surinamese	8	38%	4	19%	9	43%	21
Ethnicity	Creole	95	79%	16	13%	9	8%	120
	Maroon	37	73%	10	20%	4	8%	51
	Hindustani	33	60%	11	20%	11	20%	55
	Indigenous	26	52%	20	40%	4	8%	50
	Javanese	22	51%	7	16%	14	33%	43
	Other	31	63%	6	12%	12	24%	49
Residency	Urban	189	69%	46	17%	39	14%	274
	Non-urban	55	59%	24	26%	15	16%	94
HIV test result	Negative	199	64%	65	21%	49	15%	313
	Positive	41	80%	5	10%	5	10%	51
	Unknown	4	100%	0	0%	0	0%	4
Illicit drug use	No	164	62%	53	20%	49	18%	266
	Yes	73	79%	15	16%	5	5%	93
	Unknown	7	78%	2	22%	0	0%	9
Diabetes mellitus	No	199	68%	58	20%	37	12%	294
	Yes	39	58%	11	16%	17	25%	67
	Unknown	6	86%	1	14%	0	0%	7
Case type	New	225	65%	70	20%	53	15%	348
	Previously treated	19	95%	0	0%	1	5%	20
Disease type	Pulmonary	237	66%	69	19%	54	15%	360
	Extra-pulmonary	7	88%	1	13%	0	0%	8

Large clusters are clusters with more than six patients; small clusters are clusters with two to six patients.

The isolates of 46 of the 51 patients belonging to the second largest cluster carried a D435Y *rpoB* mutation, which is associated with low-level rifampicin resistance,^12^ whereas this mutation was absent in isolates of the remaining five patients in this cluster. Another three patients were diagnosed with rifampicin-resistant TB in other clusters, as we described previously,^19^ resulting in a total of 49 rifampicin-resistant TB cases (Table 1). Two isolates showed additional isoniazid resistance; isoniazid monoresistance was not detected by WGS.

In multivariable logistic regression analysis, indigenous ethnicity was inversely associated with membership in large clusters compared with small clusters (adjusted OR [aOR] = 0.3; 95% CI = 0.1–0.6) (Table 3). When comparing patients in large clusters with non-clustered patients, illicit drug use was three times more prevalent among individuals in large clusters (aOR = 3.0; 95% CI = 1.1–8.9). In contrast, non-Surinamese nationality (aOR = 0.2; 95% CI = 0.1–0.6), Javanese ethnicity (aOR = 0.2; 95% CI = 0.1–0.5), and other ethnicities (aOR = 0.3; 95% CI = 0.1–0.9) were independently associated with lower odds of belonging to large clusters (Table 4).

**Table 3.** Bivariate and multivariable analysis comparing patients in large and in small whole-genome sequencing clusters in Suriname, 2019–2024.

		Large versus small clusters			
Characteristics	Value	OR	95% CI	aOR	95% CI
Sex	Female	1		1	
	Male	1.1	0.6–2.1	0.7	0.4–1.5
Age (years)	<25	1		1	
	25–44	1.5	0.7–3.2	1.3	0.6–3.1
	45–64	2.3	1.0–5.2	2.1	0.9–4.9
	≥65	1.3	0.4–4.4	1.0	0.3–3.6
Nationality	Surinamese	1		^A^	
	Non-Surinamese	0.6	0.2–1.9	^A^	
Ethnicity	Creole	1		1	
	Maroon	0.6	0.3–1.5	0.7	0.3–1.7
	Hindustani	0.5	0.2–1.2	0.5	0.2–1.2
	Indigenous	0.2	0.1–0.5^B^	0.3	0.1–0.6^B^
	Javanese	0.5	0.2–1.4	0.6	0.2–1.7
	Other	0.9	0.3–2.4	0.9	0.3–2.5
Residency	Urban	1		1	
	Non-urban	0.6	0.3–1.0	0.9	0.4–1.8
HIV test result	Negative or unknown	1		1	
	Positive	2.6	1.0–6.9	1.9	0.7–5.1
Illicit drug use	No or unknown	1		1	
	Yes	1.6	0.8–2.9	1.3	0.6–2.5
Diabetes mellitus	No or unknown	1		^A^	
	Yes	1.0	0.5–2.1	^A^	

aOR = adjusted odds ratio; CI = confidence interval; OR = odds ratio.

A Apart from sex, only variables with *P* < 0.25 in bivariate were included in the multivariable analysis.

B For variables with *P* < 0.05.

**Table 4.** Bivariate and multivariable analysis comparing patients in large whole-genome sequencing clusters with non-clustered patients in Suriname, 2019–2024.

		Large versus non-clustered			
Characteristics	Value	OR	95% CI	aOR	95% CI
Sex	Female	1		1	
	Male	1.9	1.0–3.5	1.6	0.8–3.4
Age (years)	<25	1		1	
	25–44	0.7	0.2–2.4	0.8	0.2–3.2
	45–64	0.4	0.1–1.4	0.5	0.1–1.8
	≥65	0.2	0.1–0.8^B^	0.2	0.1–1.1
Nationality	Surinamese	1		1	
	Non-Surinamese	0.2	0.1–0.4	0.2	0.1–0.6^B^
Ethnicity	Creole	1		1	
	Maroon	0.9	0.2–3.0	0.7	0.2–2.7
	Hindustani	0.3	0.1–0.8^B^	0.4	0.1–1.1
	Indigenous	0.6	0.2–2.2	0.8	0.2–2.9
	Javanese	0.1	0.1–0.4^B^	0.2	0.1–0.5^B^
	Other	0.2	0.1–0.6^B^	0.3	0.1–0.9^B^
Residency	Urban	1		^A^	
	Non-urban	0.8	0.4–1.6	^A^	
HIV test result	Negative or unknown	1		1	
	Positive	2.0	0.7–5.3	1.2	0.4–3.7
Illicit drug use	No or unknown	1		1	
	Yes	4.2	1.6–10.9^B^	3.0	1.1–8.6^B^
Diabetes mellitus	No or unknown	1		1	
	Yes	0.4	0.2–0.8^B^	0.8	0.4–1.9

aOR = adjusted odds ratio; CI = confidence interval; OR = odds ratio.

A Apart from sex, only variables with *P* < 0.25 in bivariate were included in the multivariable analysis.

B For variables with *P* < 0.05.

Among the 698 patients, 658 were new patients and 40 had been previously treated (Figure). Among the new patients, 167 (25%) reported at least one presumed index patient. Of these reported index patients, 132 were recorded in the National TB Register (80 during 2019–2024 and 52 during 2010–2018), while 35 could not be identified. Forty-two had an epidemiological link to a presumed index patient whose isolate was also sequenced. Of these 42 epidemiological linked pairs, 37 (88%) had identical WGS profiles (same cluster), whereas 5 (12%) showed discordant profiles (different cluster). All five mismatched cases involved patients who had named someone belonging to a large cluster; three of these patients reported illicit drug use.

Applying the n–1 method, 13% (37/285) of clustered cases were epidemiologically linked to another case within the same cluster. Epidemiologically confirmed links were more frequently observed in small clusters than in large cluster (26% [12/47] vs. 10% [25/238], *P* < 0.05). The median time interval between treatment start dates of patients and their presumed index cases was 1.4 years for the 127 cases (interquartile range [IQR]: 0.4–3.7) with a presumed index patients identified in the National TB Register (excluding the five with discordant WGS profiles), and 1.1 years (IQR: 0.5–2.2) among the 37 genetically confirmed pairs.

## DISCUSSION

In this national WGS study, 96% of the *M. tuberculosis* isolates had a WGS result, consisting of more than 50% of the notified TB patients. In total, 83 distinct WGS profiles were identified. Sixty-six percent of patients with a WGS belonged to six large clusters (>6 patients) and 19% to small clusters (two to six patients each), and 15% had unique WGS profiles. In contrast to creoles, other ethnic groups, excluding the indigenous population (aOR = 0.3, 95% CI = 0.1–0.6), exhibited no discernible difference in their tendency to form large clusters. Illicit drug use was three times more frequently present in large clusters compared to patients with a unique WGS profiles. Epidemiologically confirmed links were more frequently observed in small clusters than in large cluster (26% vs. 10%, *P* < 0.05).

The high clustering rate (85%) in this intermediate-TB-burden country is comparable to reports from high-burden settings, such as South Africa (67%),^20^ and substantially higher than that in low-burden settings, such as Germany (25%), the UK (23%), and the Netherlands (25%) where epidemics are driven primarily by reactivation and importation.^7,21^ Furthermore, the finding that the majority of clustered patients belonged to a small number of clusters has also been reported in high-TB-burden settings, where a few dominant strains account for persistent transmission.^22^

The finding that patients who use illicit drug use were more frequently found in large clusters in Suriname is consistent with international studies demonstrating higher level of molecular clustering and ongoing transmission among people who use illicit drugs, reflecting increased exposure risk and prolonged infectious periods.^23,24^ In general, delays in marginalised subset of populations are driven by limited awareness of TB symptoms as well as substantial barriers to accessing health care services and can contribute to the formation of large genomic clusters.^23,25^ Addressing TB transmission in these population therefore requires tailored, targeted interventions, such as periodic outreach-based radiographic screening and integration of TB control activities with social services supporting marginalised populations.^26,27^ This will in Suriname not only impact TB control among illicit drug users but also reduce transmission in the wider population.

This study also provides insights into the population structure of *M. tuberculosis* in Suriname. We observed a predominance of the Euro-American lineage (lineage 4), which is consistent with findings from several Latin American countries.^28,29^ This regional pattern supports the hypothesis that European colonial expansion between the 16th and 19th centuries contributed substantially to the global dissemination of lineage 4, particularly in South America and the Caribbean.^30^ The association between indigenous ethnicity and small cluster observed in this study is consistent with findings from molecular epidemiology studies in other Latin American settings, where geographically or socially isolated populations, such as indigenous populations, tend to experience transmission within restricted contact networks, resulting in smaller, more localised clusters.^31,32^ However, transmission can also be very extensive among indigenous populations as was reported in a study from the USA among the Amerindian and Alaska Native populations.^33^

The proportion of confirmed epidemiological links in our study (13%) was substantially lower than that reported in the Netherlands (57%).^7^ This difference may have several explanations. First, cluster investigation in the Netherlands has been implemented for decades and is conducted in real time, whereas in Suriname information on strain clustering was only available retrospectively, after patients had completed treatment. Second, the Suriname NTP has very limited human resources restricting the frequency and detail of patient interviews. Third, patients may be unable to recall the index case due to the long incubation period of TB. This may be particularly relevant in large clusters that have persisted over many years. Fourth, index patients may not disclose their disease status, and this may vary across cultural contexts. Also, our results show that identifying who infected whom can be difficult and sometimes incorrect when people share complicated social environments.

Of interest, our study found that patients in small clusters were more often aware of a presumed index patient, supporting the potential value of strengthening contact investigation efforts. Furthermore, the relative short median interval between patients and their named index patient (<1.5 years) suggests that TB transmission chains may be interrupted relatively quickly if effective interventions are implemented.

This study had several strengths and limitations. A major strength was that WGS results were available for more than half of all TB patients, although this was lower in 2021 when hospitals did not fully adhere to a new diagnostic algorithm and less samples were submitted for culturing. Another strength is the high quality of the epidemiological data in the National TB Register with additional information available in the patient files. Patients interviewing and cluster investigation were suboptimal during the study period, which may have affected the proportion of confirmed transmission links. A weakness and area for further research is that the SNP thresholds to identify transmission were developed for use in a low-burden setting and may need to be interpreted differently in settings with extensive spread of closely related isolates over decades.^34^ The cluster containing both drug-resistant and drug-susceptible strains is of particular interest. A follow-up study is ongoing to investigate transmission chains using SNP-based analysis as previously described.^34^ Information on strain clustering was only available retrospectively, after patients had completed treatment, limiting its utility for real-time public health action. More recently, WGS typing results are being shared with TB nurses in a more promptly manner, enabling more detailed cluster investigation, as is routinely practiced in several other countries.^7,35^ A constraint is the severely limited manpower capacity of the National Tuberculosis Programme, which currently employs only three nurses, limiting the frequency of patient interviews, the optimal use of WGS data, and the extent of contact investigation and screening activities.

## CONCLUSION

This national WGS study provides detailed insight into *M. tuberculosis* transmission in Suriname. The high proportion of clustered cases may reflect both recent transmission and long-term circulation of related strains. Illicit drug use was associated with membership in large transmission cluster, highlighting the need for active case finding, in collaboration with the substance-use services. The presence of smaller clusters suggests more localised community transmission, and underscores the need for strengthened contact investigation. These activities are essential to interrupt TB transmission in Suriname and accelerate progress towards the WHO End TB target of reducing the TB burden.

## Supplementary Material
