# Supplementary material for: Genomic surveillance of TB: cluster identification and transmission insights
Source: IJTLD Open. 2026 Nov 13;3(11):714–21. doi: 10.5588/ijtldopen.26.0115 (PMC13763967; doi:10.5588/ijtldopen.26.0115)
Supplement: Supplementary file 1 [file ijtldopen26-0115_supplementarydata1.pdf]

Supplementary tables

Genomic surveillance of tuberculosis in Suriname: cluster identification and transmission insights.

E. Commiesie, R. Anthony, F. Gopie, M. Kamst, T. Koendjbiharie, S. Sewdien, D. Stijnberg, S. Vreden, R. De Zwaan, G. de Vries

Supplementary table 1. Details of lineages, sublineages, WGS fingerprint, cluster sizes, mutations, and sample accession numbers of the 368 *Mycobacterium tuberculosis* isolates included in this study

ID	Lineage	Sublineage	WGS fingerprint	Cluster size	Mutation	Sample Accession Number
2019-27	lineage1	lineage1.1.1	SUR10	Unique WGS		ERR17720229
2020-8	lineage1	lineage1.1.2	SUR30	Unique WGS		ERR17720275
2021-13	lineage1	lineage1.1.2	SUR43	Small		ERR17720332
2022-7	lineage1	lineage1.1.2	SUR43	Small		ERR17720356
2024-6	lineage1	lineage1.1.2	SUR77	Unique WGS		ERR17720511
2019-1	lineage1	lineage1.1.3	SUR01	Large	rpoB_D435Y, katG_INS & ahpC_g-51a *	ERR17720271
2019-5	lineage1	lineage1.1.3	SUR01	Large	rpoB_D435Y	ERR17720205
2019-14	lineage1	lineage1.1.3	SUR01	Large	rpoB_D435Y	ERR17720213
2019-25	lineage1	lineage1.1.3	SUR01	Large	rpoB_D435Y	ERR17720226
2019-29	lineage1	lineage1.1.3	SUR01	Large	rpoB_D435Y	ERR17720232
2019-33	lineage1	lineage1.1.3	SUR01	Large	rpoB_D435Y	ERR17720233
2019-38	lineage1	lineage1.1.3	SUR01	Large	rpoB_D435Y	ERR17720230
2019-53	lineage1	lineage1.1.3	SUR01	Large	rpoB_D435Y	ERR17720237
2019-60	lineage1	lineage1.1.3	SUR01	Large	rpoB_D435Y	ERR17720260
2019-66	lineage1	lineage1.1.3	SUR01	Large	rpoB_D435Y	ERR17720261
2020-2	lineage1	lineage1.1.3	SUR01	Large	rpoB_D435Y	ERR17720272
2020-20	lineage1	lineage1.1.3	SUR01	Large	rpoB_D435Y	ERR17720283
2020-22	lineage1	lineage1.1.3	SUR01	Large	rpoB_D435Y	ERR17720284
2020-42	lineage1	lineage1.1.3	SUR01	Large	rpoB_D435Y	ERR17720312

2020-50	lineage1	lineage1.1.3	SUR01	Large	rpoB_D435Y	ERR17720317
2020-52	lineage1	lineage1.1.3	SUR01	Large	rpoB_D435Y	ERR17720319
2021-3	lineage1	lineage1.1.3	SUR01	Large	rpoB_D435Y	ERR17720324
2021-6	lineage1	lineage1.1.3	SUR01	Large	rpoB_D435Y	ERR17720323
2021-14	lineage1	lineage1.1.3	SUR01	Large	rpoB_D435Y	ERR17720334
2021-26	lineage1	lineage1.1.3	SUR01	Large	rpoB_D435Y	ERR17720345
2021-27	lineage1	lineage1.1.3	SUR01	Large	rpoB_D435Y	ERR17720346
2022-2	lineage1	lineage1.1.3	SUR01	Large	rpoB_D435Y	ERR17720351
2022-5	lineage1	lineage1.1.3	SUR01	Large		ERR17720355
2022-14	lineage1	lineage1.1.3	SUR01	Large	rpoB_D435Y	ERR17720362
2022-23	lineage1	lineage1.1.3	SUR01	Large		ERR17720374
2022-34	lineage1	lineage1.1.3	SUR01	Large	rpoB_D435Y	ERR17720383
2022-60	lineage1	lineage1.1.3	SUR01	Large		ERR17720391
2022-64	lineage1	lineage1.1.3	SUR01	Large	rpoB_D435Y	ERR17720413
2022-68	lineage1	lineage1.1.3	SUR01	Large	rpoB_D435Y	ERR17720420
2022-70	lineage1	lineage1.1.3	SUR01	Large	rpoB_D435Y	ERR17720416
2023-19	lineage1	lineage1.1.3	SUR01	Large	rpoB_D435Y	ERR17720447
2023-27	lineage1	lineage1.1.3	SUR01	Large	rpoB_D435Y	ERR17720457
2023-32	lineage1	lineage1.1.3	SUR01	Large	rpoB_D435Y	ERR17720460
2023-42	lineage1	lineage1.1.3	SUR01	Large	rpoB_D435Y	ERR17720468
2023-47	lineage 1/4	lineage1.1.3 / 4.4.2	SUR01 / SUR03 ‡	Large	rpoB_D435Y	ERR17720476
2023-49	lineage1	lineage1.1.3	SUR01	Large	rpoB_D435Y	ERR17720478
2023-51	lineage1	lineage1.1.3	SUR01	Large	rpoB_D435Y	ERR17720482
2023-52	lineage1	lineage1.1.3	SUR01	Large	rpoB_D435Y	ERR17720483
2023-55	lineage1	lineage1.1.3	SUR01	Large	rpoB_D435Y	ERR17720487
2023-70	lineage1	lineage1.1.3	SUR01	Large	rpoB_D435Y	ERR17720500
2023-72	lineage1	lineage1.1.3	SUR01	Large	rpoB_D435Y	ERR17720503
2024-10	lineage1	lineage1.1.3	SUR01	Large	rpoB_D435Y	ERR17720512
2024-11	lineage1	lineage1.1.3	SUR01	Large		ERR17720513
2024-16	lineage1	lineage1.1.3	SUR01	Large	rpoB_D435Y	ERR17720521

2024-22	lineage1	lineage1.1.3	SUR01	Large	rpoB_D435Y	ERR17720526
2024-29	lineage1	lineage1.1.3	SUR01	Large	rpoB_D435Y	ERR17720535
2024-37	lineage1	lineage1.1.3	SUR01	Large	rpoB_D435Y	ERR17720542
2024-43	lineage1	lineage1.1.3	SUR01	Large	rpoB_D435Y	ERR17720548
2024-46	lineage1	lineage1.1.3	SUR01	Large		ERR17720549
2024-54	lineage1	lineage1.1.3	SUR01	Large	rpoB_D435Y	ERR17720559
2024-56	lineage1	lineage1.1.3	SUR01	Large	rpoB_D435Y	ERR17720561
2019-49	lineage1	lineage1.1.3	SUR16	Unique WGS		ERR17720246
2020-6	lineage1	lineage1.1.3	SUR28	Unique WGS		ERR17720273
2020-19	lineage1	lineage1.1.3	SUR31	Unique WGS		ERR17720282
2020-30	lineage1	lineage1.1.3	SUR38	Unique WGS		ERR17720297
2022-17	lineage1	lineage1.1.3	SUR49	Unique WGS		ERR17720366
2022-67	lineage1	lineage1.1.3	SUR61	Small		ERR17720419
2023-8	lineage1	lineage1.1.3	SUR61	Small		ERR17720439
2023-73	lineage1	lineage1.1.3	SUR71	Unique WGS		ERR17720504
2024-34	lineage1	lineage1.1.3	SUR82	Unique WGS		ERR17720538
2019-16	lineage1	lineage1.2.1	SUR05	Small		ERR17720221
2020-31	lineage1	lineage1.2.1	SUR05	Small		ERR17720299
2023-34	lineage1	lineage1.2.1	SUR05	Small		ERR17720461
2023-44	lineage1	lineage1.2.1	SUR05	Small		ERR17720473
2019-8	lineage1	lineage1.2.1	SUR20	Large		ERR17720203
2019-64	lineage1	lineage1.2.1	SUR20	Large		ERR17720262
2020-13	lineage1	lineage1.2.1	SUR20	Large		ERR17720280
2020-36	lineage1	lineage1.2.1	SUR20	Large		ERR17720304
2021-2	lineage1	lineage1.2.1	SUR20	Large		ERR17720322
2022-32	lineage1	lineage1.2.1	SUR20	Large		ERR17720381
2022-48	lineage1	lineage1.2.1	SUR20	Large		ERR17720398
2022-62	lineage1	lineage1.2.1	SUR20	Large		ERR17720411
2023-17	lineage1	lineage1.2.1	SUR20	Large		ERR17720449
2023-31	lineage1	lineage1.2.1	SUR20	Large		ERR17720462

2024-9	lineage1	lineage1.2.1	SUR20	Large		ERR17720515
2020-11	lineage1	lineage1.2.1	SUR33	Unique WGS		ERR17720279
2021-16	lineage1	lineage1.2.1	SUR45	Unique WGS		ERR17720337
2022-57	lineage1	lineage1.2.1	SUR56	Unique WGS		ERR17720408
2023-20	lineage1	lineage1.2.1	SUR65	Unique WGS	rpoB_D435Y, fabG1 t-8c and inhAI21V	ERR17720446
2019-28	lineage1	lineage1.2.2	SUR09	Unique WGS		ERR17720228
2019-69	lineage1	lineage1.2.2	SUR21	Small		ERR17720267
2023-2	lineage1	lineage1.2.2	SUR21	Small		ERR17720430
2023-62	lineage1	lineage1.2.2	SUR21	Small		ERR17720492
2024-57	lineage1	lineage1.2.2	SUR21	Small		ERR17720563
2020-33	lineage1	lineage1.2.2	SUR39	Unique WGS		ERR17720300
2022-24	lineage1	lineage1.2.2	SUR50	Unique WGS		ERR17720372
2024-4	lineage1	lineage1.2.2	SUR75	Small		ERR17720509
2024-61	lineage1	lineage1.2.2	SUR75	Small		ERR17720568
2024-5	lineage1	lineage1.2.2	SUR76	Unique WGS		ERR17720510
2024-12	lineage1	lineage1.2.2	SUR78	Unique WGS		ERR17720517
2024-40	lineage1	lineage1.2.2	SUR84	Small		ERR17720544
2024-48	lineage1	lineage1.2.2	SUR84	Small		ERR17720553
2019-19	lineage2	lineage2.2.1	SUR02	Small		ERR17720216
2019-42	lineage2	lineage2.2.1	SUR02	Small		ERR17720239
2021-11	lineage2	lineage2.2.1	SUR02	Small		ERR17720330
2019-21	lineage2	lineage2.2.1	SUR04	Unique WGS	rpsl_K43R	ERR17720220
2019-9	lineage2	lineage2.2.1	SUR06	Large		ERR17720208
2019-10	lineage2	lineage2.2.1	SUR06	Large		ERR17720209
2019-22	lineage2	lineage2.2.1	SUR06	Large		ERR17720222
2019-51	lineage2	lineage2.2.1	SUR06	Large		ERR17720254
2019-57	lineage2	lineage2.2.1	SUR06	Large		ERR17720255
2020-28	lineage2	lineage2.2.1	SUR06	Large		ERR17720296
2020-35	lineage2	lineage2.2.1	SUR06	Large		ERR17720303

2020-39	lineage2	lineage2.2.1	SUR06	Large		ERR17720308
2020-40	lineage2	lineage2.2.1	SUR06	Large		ERR17720307
2021-12	lineage2	lineage2.2.1	SUR06	Large		ERR17720331
2021-20	lineage2	lineage2.2.1	SUR06	Large		ERR17720310
2022-22	lineage2	lineage2.2.1	SUR06	Large		ERR17720371
2022-45	lineage2	lineage2.2.1	SUR06	Large		ERR17720396
2022-53	lineage2	lineage2.2.1	SUR06	Large		ERR17720403
2022-55	lineage2	lineage2.2.1	SUR06	Large		ERR17720406
2022-74	lineage2	lineage2.2.1	SUR06	Large		ERR17720426
2023-5	lineage2	lineage2.2.1	SUR06	Large		ERR17720437
2023-29	lineage2	lineage2.2.1	SUR06	Large		ERR17720458
2023-56	lineage2	lineage2.2.1	SUR06	Large		ERR17720485
2024-2	lineage2	lineage2.2.1	SUR06	Large		ERR17720508
2024-17	lineage2	lineage2.2.1	SUR06	Large		ERR17720523
2024-55	lineage2	lineage2.2.1	SUR06	Large		ERR17720565
2023-3	lineage2	lineage2.2.1	SUR60	Small		ERR17720433
2023-36	lineage2	lineage2.2.1	SUR60	Small		ERR17720463
2024-19	lineage2	lineage2.2.1	SUR60	Small		ERR17720522
2023-10	lineage2	lineage2.2.1	SUR63	Small		ERR17720438
2024-23	lineage2	lineage2.2.1	SUR63	Small		ERR17720527
2023-38	lineage2	lineage2.2.1	SUR68	Small		ERR17720467
2024-30	lineage2	lineage2.2.1	SUR68	Small		ERR17720536
2023-54	lineage2	lineage2.2.1	SUR70	Unique WGS		ERR17720484
2023-66	lineage2	lineage2.2.1	SUR72	Unique WGS		ERR17720496
2024-20	lineage2	lineage2.2.1	SUR81	Unique WGS		ERR17720525
2023-12	lineage2	lineage2.2.1.1	SUR64	Unique WGS		ERR17720441
2024-27	lineage2	lineage2.2.1.1	SUR80	Unique WGS		ERR17720532
2019-11	lineage3	lineage3	SUR24	Small		ERR17720211
2022-8	lineage3	lineage3	SUR24	Small		ERR17720357
2022-4	lineage3	lineage3	SUR48	Unique WGS		ERR17720353

2022-30	lineage3	lineage3	SUR52	Unique WGS		ERR17720378
2019-31	lineage4	lineage4	SUR12	Small		ERR17720558
2022-31	lineage4	lineage4	SUR12	Small		ERR17720380
2024-21	lineage4	lineage4	SUR12	Small		ERR17720524
2020-29	lineage4	lineage4	SUR37	Small		ERR17720295
2021-22	lineage4	lineage4	SUR37	Small		ERR17720340
2023-63	lineage4	lineage4	SUR37	Small		ERR17720493
2024-38	lineage4	lineage4	SUR37	Small		ERR17720545
2019-65	lineage4	lineage4	SUR47	Small		ERR17720263
2022-1	lineage4	lineage4	SUR47	Small		ERR17720350
2022-3	lineage4	lineage4	SUR47	Small		ERR17720352
2023-6	lineage4	lineage4	SUR47	Small		ERR17720434
2023-69	lineage4	lineage4	SUR47	Small		ERR17720498
2022-36	lineage4	lineage4	SUR53	Unique WGS		ERR17720384
2019-45	lineage4	lineage4.1	SUR15	Small		ERR17720245
2019-62	lineage4	lineage4.1	SUR15	Small		ERR17720264
2020-34	lineage4	lineage4.1	SUR15	Small		ERR17720301
2021-28	lineage4	lineage4.1	SUR15	Small		ERR17720347
2019-63	lineage4	lineage4.1.2	SUR73	Unique WGS		ERR17720258
2024-1	lineage4	lineage4.1.2	SUR74	Unique WGS		ERR17720506
2019-56	lineage4	lineage4.1.2.1	SUR18	Small		ERR17720253
2022-13	lineage4	lineage4.1.2.1	SUR18	Small		ERR17720361
2023-16	lineage4	lineage4.1.2.1	SUR18	Small		ERR17720436
2023-59	lineage4	lineage4.1.2.1	SUR18	Small		ERR17720488
2020-7	lineage4	lineage4.1.2.1	SUR29	Small		ERR17720274
2020-10	lineage4	lineage4.1.2.1	SUR29	Small		ERR17720292
2020-25	lineage4	lineage4.1.2.1	SUR29	Small		ERR17720289
2020-18	lineage4	lineage4.1.2.1	SUR32	Small		ERR17720288
2020-21	lineage4	lineage4.1.2.1	SUR32	Small		ERR17720285
2023-30	lineage4	lineage4.1.2.1	SUR32	Small		ERR17720431

2023-45	lineage4	lineage4.1.2.1	SUR32	Small		ERR17720477
2020-26	lineage4	lineage4.1.2.1	SUR36	Small		ERR17720293
2021-18	lineage4	lineage4.1.2.1	SUR36	Small		ERR17720339
2021-5	lineage4	lineage4.1.2.1	SUR41	Unique WGS		ERR17720325
2021-8	lineage4	lineage4.1.2.1	SUR42	Unique WGS		ERR17720329
2022-75	lineage4	lineage4.1.2.1	SUR79	Unique WGS	rpoB_Q432E	ERR17720501
2022-28	lineage4	lineage4.3.1	SUR51	Unique WGS		ERR17720376
2019-30	lineage4	lineage4.3.2	SUR11	Unique WGS		ERR17720231
2019-47	lineage4	lineage4.3.3	SUR17	Unique WGS		ERR17720248
2020-4	lineage4	lineage4.3.3	SUR27	Unique WGS		ERR17720270
2020-24	lineage4	lineage4.3.3	SUR34	Small		ERR17720287
2022-71	lineage4	lineage4.3.3	SUR34	Small		ERR17720418
2020-48	lineage4	lineage4.3.3	SUR40	Unique WGS		ERR17720316
2022-56	lineage4	lineage4.3.3	SUR55	Small		ERR17720405
2024-26	lineage4	lineage4.3.3	SUR55	Small		ERR17720529
2024-41	lineage4	lineage4.3.3	SUR55	Small		ERR17720546
2023-1	lineage4	lineage4.3.3	SUR59	Unique WGS		ERR17720429
2024-62	lineage4	lineage4.3.3	SUR85	Unique WGS		ERR17720555
2019-12	lineage4	lineage4.3.4.1	SUR25	Unique WGS		ERR17720212
2021-21	lineage4	lineage4.3.4.1	SUR46	Small		ERR17720341
2024-44	lineage4	lineage4.3.4.1	SUR46	Small		ERR17720551
2023-15	lineage4	lineage4.3.4.1	SUR66	Unique WGS		ERR17720443
2019-40	lineage4	lineage4.3.4.2	SUR14	Unique WGS		ERR17720241
2019-58	lineage4	lineage4.3.4.2	SUR19	Unique WGS		ERR17720256
2019-59	lineage4	lineage4.3.4.2	SUR26	Unique WGS		ERR17720259
2023-28	lineage4	lineage4.4.1.1	SUR67	Unique WGS		ERR17720456
2023-41	lineage4	lineage4.4.1.1	SUR69	Unique WGS		ERR17720470
2019-4	lineage4	lineage4.4.2	SUR03	Large		ERR17720201
2019-7	lineage4	lineage4.4.2	SUR03	Large		ERR17720206
2019-13	lineage4	lineage4.4.2	SUR03	Large		ERR17720210

2019-20	lineage4	lineage4.4.2	SUR03	Large		ERR17720219
2019-32	lineage4	lineage4.4.2	SUR03	Large		ERR17720217
2019-43	lineage4	lineage4.4.2	SUR03	Large		ERR17720243
2019-44	lineage4	lineage4.4.2	SUR03	Large		ERR17720244
2019-46	lineage4	lineage4.4.2	SUR03	Large		ERR17720247
2019-52	lineage4	lineage4.4.2	SUR03	Large		ERR17720251
2019-54	lineage4	lineage4.4.2	SUR03	Large		ERR17720252
2019-55	lineage4	lineage4.4.2	SUR03	Large		ERR17720224
2019-67	lineage4	lineage4.4.2	SUR03	Large		ERR17720265
2019-68	lineage4	lineage4.4.2	SUR03	Large		ERR17720266
2020-1	lineage4	lineage4.4.2	SUR03	Large		ERR17720268
2020-12	lineage4	lineage4.4.2	SUR03	Large		ERR17720277
2020-37	lineage4	lineage4.4.2	SUR03	Large		ERR17720305
2020-38	lineage4	lineage4.4.2	SUR03	Large		ERR17720306
2020-45	lineage4	lineage4.4.2	SUR03	Large		ERR17720309
2020-51	lineage4	lineage4.4.2	SUR03	Large		ERR17720318
2021-15	lineage4	lineage4.4.2	SUR03	Large		ERR17720335
2021-17	lineage4	lineage4.4.2	SUR03	Large		ERR17720336
2021-24	lineage4	lineage4.4.2	SUR03	Large		ERR17720343
2022-6	lineage4	lineage4.4.2	SUR03	Large	rpoB H445R	ERR17720354
2022-12	lineage4	lineage4.4.2	SUR03	Large		ERR17720364
2022-16	lineage4	lineage4.4.2	SUR03	Large		ERR17720365
2022-20	lineage4	lineage4.4.2	SUR03	Large		ERR17720368
2022-39	lineage4	lineage4.4.2	SUR03	Large		ERR17720388
2022-47	lineage4	lineage4.4.2	SUR03	Large		ERR17720395
2022-51	lineage4	lineage4.4.2	SUR03	Large		ERR17720401
2022-59	lineage4	lineage4.4.2	SUR03	Large		ERR17720407
2022-61	lineage4	lineage4.4.2	SUR03	Large		ERR17720410
2022-78	lineage4	lineage4.4.2	SUR03	Large		ERR17720424
2022-80	lineage4	lineage4.4.2	SUR03	Large		ERR17720428

2023-11	lineage4	lineage4.4.2	SUR03	Large		ERR17720440
2023-13	lineage4	lineage4.4.2	SUR03	Large		ERR17720445
2023-23	lineage4	lineage4.4.2	SUR03	Large		ERR17720452
2023-50	lineage4	lineage4.4.2	SUR03	Large		ERR17720479
2023-64	lineage4	lineage4.4.2	SUR03	Large		ERR17720494
2023-65	lineage4	lineage4.4.2	SUR03	Large		ERR17720495
2024-18	lineage4	lineage4.4.2	SUR03	Large		ERR17720528
2023-75	lineage4	lineage4.4.2	SUR03	Large		ERR17720474
2024-25	lineage4	lineage4.4.2	SUR03	Large		ERR17720531
2024-52	lineage4	lineage4.4.2	SUR03	Large		ERR17720564
2021-10	lineage4	lineage4.4.2	SUR44	Unique WGS		ERR17720333
2022-41	lineage4	lineage4.4.2	SUR54	Unique WGS		ERR17720393
2023-4	lineage4	lineage4.5	SUR62	Unique WGS		ERR17720432
2022-72	lineage4	lineage4.7	SUR58	Unique WGS		ERR17720422
2019-2	lineage4	lineage4.8	SUR07	Large		ERR17720204
2019-3	lineage4	lineage4.8	SUR07	Large		ERR17720202
2019-15	lineage4	lineage4.8	SUR07	Large		ERR17720214
2019-17	lineage4	lineage4.8	SUR07	Large		ERR17720215
2019-18	lineage4	lineage4.8	SUR07	Large		ERR17720218
2019-23	lineage4	lineage4.8	SUR07	Large		ERR17720223
2019-26	lineage4	lineage4.8	SUR07	Large		ERR17720227
2019-34	lineage4	lineage4.8	SUR07	Large		ERR17720235
2019-35	lineage4	lineage4.8	SUR07	Large		ERR17720234
2019-37	lineage4	lineage4.8	SUR07	Large		ERR17720240
2019-48	lineage4	lineage4.8	SUR07	Large		ERR17720250
2020-3	lineage4	lineage4.8	SUR07	Large		ERR17720269
2020-5	lineage4	lineage4.8	SUR07	Large		ERR17720455
2020-9	lineage4	lineage4.8	SUR07	Large		ERR17720276
2020-16	lineage4	lineage4.8	SUR07	Large		ERR17720281
2020-23	lineage4	lineage4.8	SUR07	Large		ERR17720286

2020-27	lineage4	lineage4.8	SUR07	Large		ERR17720294
2020-32	lineage4	lineage4.8	SUR07	Large		ERR17720298
2020-41	lineage4	lineage4.8	SUR07	Large		ERR17720302
2020-43	lineage4	lineage4.8	SUR07	Large		ERR17720328
2020-44	lineage4	lineage4.8	SUR07	Large		ERR17720311
2020-46	lineage4	lineage4.8	SUR07	Large		ERR17720314
2020-47	lineage4	lineage4.8	SUR07	Large		ERR17720315
2020-49	lineage4	lineage4.8	SUR07	Large		ERR17720313
2021-1	lineage4	lineage4.8	SUR07	Large		ERR17720320
2021-9	lineage4	lineage4.8	SUR07	Large		ERR17720327
2021-19	lineage4	lineage4.8	SUR07	Large		ERR17720338
2021-23	lineage4	lineage4.8	SUR07	Large		ERR17720342
2021-25	lineage4	lineage4.8	SUR07	Large		ERR17720344
2021-29	lineage4	lineage4.8	SUR07	Large		ERR17720348
2021-30	lineage4	lineage4.8	SUR07	Large		ERR17720349
2022-9	lineage4	lineage4.8	SUR07	Large		ERR17720359
2022-10	lineage4	lineage4.8	SUR07	Large		ERR17720360
2022-11	lineage4	lineage4.8	SUR07	Large		ERR17720358
2022-15	lineage4	lineage4.8	SUR07	Large		ERR17720363
2022-18	lineage4	lineage4.8	SUR07	Large		ERR17720367
2022-19	lineage4	lineage4.8	SUR07	Large		ERR17720369
2022-21	lineage4	lineage4.8	SUR07	Large		ERR17720370
2022-26	lineage4	lineage4.8	SUR07	Large		ERR17720375
2022-29	lineage4	lineage4.8	SUR07	Large		ERR17720379
2022-33	lineage4	lineage4.8	SUR07	Large		ERR17720382
2022-35	lineage4	lineage4.8	SUR07	Large		ERR17720385
2022-37	lineage4	lineage4.8	SUR07	Large		ERR17720386
2022-38	lineage4	lineage4.8	SUR07	Large		ERR17720387
2022-40	lineage4	lineage4.8	SUR07	Large		ERR17720390
2022-42	lineage4	lineage4.8	SUR07	Large		ERR17720389

2022-43	lineage4	lineage4.8	SUR07	Large		ERR17720392
2022-44	lineage4	lineage4.8	SUR07	Large		ERR17720394
2022-46	lineage4	lineage4.8	SUR07	Large		ERR17720397
2022-49	lineage4	lineage4.8	SUR07	Large		ERR17720400
2022-52	lineage4	lineage4.8	SUR07	Large		ERR17720404
2022-54	lineage4	lineage4.8	SUR07	Large		ERR17720402
2022-58	lineage4	lineage4.8	SUR07	Large		ERR17720409
2022-63	lineage4	lineage4.8	SUR07	Large		ERR17720412
2022-65	lineage4	lineage4.8	SUR07	Large		ERR17720414
2022-66	lineage4	lineage4.8	SUR07	Large		ERR17720415
2022-69	lineage4	lineage4.8	SUR07	Large		ERR17720417
2022-76	lineage4	lineage4.8	SUR07	Large		ERR17720423
2023-7	lineage4	lineage4.8	SUR07	Large		ERR17720435
2023-9	lineage4	lineage4.8	SUR07	Large		ERR17720444
2023-14	lineage4	lineage4.8	SUR07	Large		ERR17720442
2023-18	lineage4	lineage4.8	SUR07	Large		ERR17720448
2023-21	lineage4	lineage4.8	SUR07	Large		ERR17720450
2023-22	lineage4	lineage4.8	SUR07	Large		ERR17720451
2023-24	lineage4	lineage4.8	SUR07	Large		ERR17720453
2023-25	lineage4	lineage4.8	SUR07	Large		ERR17720459
2023-26	lineage4	lineage4.8	SUR07	Large		ERR17720454
2023-35	lineage4	lineage4.8	SUR07	Large		ERR17720464
2023-37	lineage4	lineage4.8	SUR07	Large		ERR17720466
2023-43	lineage4	lineage4.8	SUR07	Large		ERR17720472
2023-46	lineage4	lineage4.8	SUR07	Large		ERR17720475
2023-53	lineage4	lineage4.8	SUR07	Large		ERR17720481
2023-57	lineage4	lineage4.8	SUR07	Large		ERR17720486
2023-58	lineage4	lineage4.8	SUR07	Large		ERR17720490
2023-60	lineage4	lineage4.8	SUR07	Large		ERR17720489
2023-67	lineage4	lineage4.8	SUR07	Large		ERR17720497

2023-68	lineage4	lineage4.8	SUR07	Large		ERR17720499
2023-71	lineage4	lineage4.8	SUR07	Large		ERR17720502
2024-3	lineage4	lineage4.8	SUR07	Large		ERR17720507
2024-7	lineage4	lineage4.8	SUR07	Large		ERR17720514
2024-8	lineage4	lineage4.8	SUR07	Large		ERR17720516
2024-13	lineage4	lineage4.8	SUR07	Large		ERR17720518
2024-15	lineage4	lineage4.8	SUR07	Large		ERR17720519
2024-24	lineage4	lineage4.8	SUR07	Large		ERR17720530
2024-31	lineage4	lineage4.8	SUR07	Large		ERR17720533
2024-32	lineage4	lineage4.8	SUR07	Large		ERR17720537
2024-33	lineage4	lineage4.8	SUR07	Large		ERR17720540
2024-35	lineage4	lineage4.8	SUR07	Large		ERR17720539
2024-36	lineage4	lineage4.8	SUR07	Large		ERR17720541
2024-42	lineage4	lineage4.8	SUR07	Large		ERR17720547
2024-45	lineage4	lineage4.8	SUR07	Large		ERR17720560
2024-47	lineage4	lineage4.8	SUR07	Large		ERR17720552
2024-49	lineage4	lineage4.8 / 4.4.2	SUR07 / SUR03 †	Large		ERR17720491
2024-50	lineage4	lineage4.8	SUR07	Large		ERR17720557
2024-51	lineage4	lineage4.8	SUR07	Large		ERR17720556
2024-53	lineage4	lineage4.8	SUR07	Large		ERR17720550
2024-59	lineage4	lineage4.8	SUR07	Large		ERR17720567
2024-60	lineage4	lineage4.8	SUR07	Large		ERR17720566
2019-6	lineage4	lineage4.8	SUR08	Large		ERR17720207
2019-24	lineage4	lineage4.8	SUR08	Large		ERR17720225
2019-39	lineage4	lineage4.8	SUR08	Large		ERR17720238
2019-41	lineage4	lineage4.8	SUR08	Large		ERR17720242
2020-14	lineage4	lineage4.8	SUR08	Large		ERR17720278
2020-15	lineage4	lineage4.8	SUR08	Large		ERR17720291
2021-4	lineage4	lineage4.8	SUR08	Large		ERR17720321
2022-25	lineage4	lineage4.8	SUR08	Large		ERR17720373

2022-27	lineage4	lineage4.8	SUR08	Large		ERR17720377
2022-50	lineage4	lineage4.8	SUR08	Large		ERR17720399
2022-77	lineage4	lineage4.8	SUR08	Large		ERR17720425
2022-79	lineage4	lineage4.8	SUR08	Large		ERR17720427
2023-33	lineage4	lineage4.8	SUR08	Large		ERR17720465
2023-39	lineage4	lineage4.8	SUR08	Large		ERR17720471
2023-40	lineage4	lineage4.8	SUR08	Large		ERR17720469
2023-61	lineage4	lineage4.8	SUR08	Large		ERR17720554
2023-74	lineage4	lineage4.8	SUR08	Large		ERR17720505
2024-28	lineage4	lineage4.8	SUR08	Large		ERR17720534
2024-58	lineage4	lineage4.8	SUR08	Large		ERR17720562
2019-36	lineage4	lineage4.8	SUR13	Small		ERR17720236
2019-50	lineage4	lineage4.8	SUR13	Small		ERR17720249
2019-61	lineage4	lineage4.8	SUR13	Small		ERR17720257
2021-7	lineage4	lineage4.8	SUR13	Small		ERR17720326
2023-48	lineage4	lineage4.8	SUR13	Small		ERR17720480
2024-14	lineage4	lineage4.8	SUR13	Small		ERR17720520
2020-17	lineage4	lineage4.8	SUR35	Unique WGS		ERR17720290
2022-73	lineage4	lineage4.8	SUR57	Unique WGS		ERR17720421
2024-39	lineage4	lineage4.8	SUR83	Unique WGS		ERR17720543

Samples are uploaded to European Nucleotide Archive, under study number PRJEB123311. Full dataset accessible via https://www.ebi.ac.uk/ena/browser/view/PRJEB123311.

[*] Patient had 3 episodes of TB. The first isolate had a rpoB_D435Y mutation, the 2nd and 3rd isolate the an additional katG_INS & ahpC_g-51a mutation.

[‡] Mixed infection. Lineage1.1.3 / SUR01 was selected as the primary isolate for further analysis because of its drug-resistant phenotype.

[†] Mixed infection. Lineage4.8 / SUR07 was selected as the primary isolate for further analysis due to its predominance in the sample.

Supplementary table 2. Summary of lineages, sublineages, and WGS fingerprints of the 368 *Mycobacterium tuberculosis* isolates included in this study.

Lineage	(sub)lineage	WGS fingerprints	Total number of isolates	Remark
**Lineage 1**	lineage1.1.1	SUR10	1	
(Indo-Oceanic)	lineage1.1.2	SUR30	1	
		SUR43	2	
		SUR77	1	
	lineage1.1.3	SUR01	51	45x Rif R (rpoB_D435Y), 1x MDR (rpoB_D435Y, fabG1 t-8c and inhAI21V)
		SUR16	1	
		SUR28	1	
		SUR31	1	
		SUR38	1	
		SUR49	1	
		SUR61	2	
		SUR71	1	
		SUR82	1	
	lineage1.2.1	SUR05	4	
		SUR20	11	
		SUR33	1	
		SUR45	1	
		SUR56	1	
		SUR65	1	rpoB_D435Y, fabG1 t-8c and inhAI21V
	lineage1.2.2	SUR09	1	
		SUR21	4	
		SUR39	1	
		SUR50	1	
		SUR75	2	
		SUR76	1	

		SUR78	1	
		SUR84	2	
	**Total lineage 1**		**97**	**26%**
**Lineage 2** (Beijing)	lineage2.2.1	SUR02	3	
		SUR04	1	
		SUR06	22	
		SUR60	3	
		SUR63	2	
		SUR68	2	
		SUR70	1	
		SUR72	1	
		SUR81	1	
	lineage2.2.1.1	SUR64	1	
		SUR80	1	
	**Total lineage 2**		**38**	**10%**
**Lineage 3** (East-African-Indian)	lineage3	SUR24	2	
		SUR48	1	
		SUR52	1	
	**Total lineage 3**		**4**	**1%**
**Lineage 4** (Euro-American)	lineage4	SUR12	3	
		SUR37	4	
		SUR47	5	
		SUR53	1	
	lineage4.1	SUR15	4	
	lineage4.1.2	SUR73	1	
		SUR74	1	
	lineage4.1.2.1	SUR18	4	
		SUR29	3	
		SUR32	4	

	SUR36	2	
	SUR41	1	
	SUR42	1	
	SUR79	1	Rif mut (rpoB_Q432E)
lineage4.3.1	SUR51	1	
lineage4.3.2	SUR11	1	
lineage4.3.3	SUR17	1	
	SUR27	1	
	SUR34	2	
	SUR40	1	
	SUR55	3	
	SUR59	1	
	SUR85	1	
lineage4.3.4.1	SUR25	1	
	SUR46	2	
	SUR66	1	
lineage4.3.4.2	SUR14	1	
	SUR19	1	
	SUR26	1	
lineage4.4.1.1	SUR67	1	
	SUR69	1	
lineage4.4.2	SUR03	43	1x Rif R (rpoB_H445R)
	SUR44	1	
	SUR54	1	
lineage4.5	SUR62	1	
lineage4.7	SUR58	1	
lineage4.8	SUR07	98	
	SUR08	19	
	SUR13	6	
	SUR35	1	

		SUR57	1	
		SUR83	1	
	**Total lineage 4**		**229**	**62%**
**Total**		**83**	**368**	
